# The Future of the Monograph in the Arts, Humanities and Social Sciences: Publisher Perspectives on a Transitioning Format

**DOI:** 10.1007/s12109-023-09937-1

**Published:** 2023-02-22

**Authors:** Philip Shaw, Angus Phillips, Maria Bajo Gutiérrez

**Affiliations:** Oxford International Centre for Publishing, Oxford Brookes University, Headington Hill Hall, Oxford, OX3 0BP UK

**Keywords:** Monograph, AHSS, Open Access, Metadata, Creative Commons, Scholarly communication

## Abstract

A web-based survey of academic publishers was undertaken in 2021 by a team at Oxford International Centre for Publishing into the state of monograph publication in the arts, humanities, and social sciences. 25 publishing organisations responded, including many of the larger presses, representing approximately 75% of monograph output. Responses to the survey showed that the Covid 19 pandemic has accelerated the existing trend from print to digital dissemination and that Open Access (OA) titles receive substantially greater levels of usage than those published traditionally. Responses also showed that for most publishers OA publication stands at under 25% of output and that fewer than 10% of authors enquire about OA publication options. Continuing problem areas highlighted by respondents were the clearing of rights for OA publication and the standardisation of title and usage metadata. All responding organisations confirmed that they expect to be publishing monographs in ten years’ time, but that they anticipate the format and/or the model will be different, with open access expected to play a key part in the future, perhaps in the context of a mixed economy of OA and ‘toll access’ publication.

## Introduction

A web survey was conducted into the state of monograph publishing in 2021 from the perspective of publishers, both commercial and university presses. The survey was undertaken at a time when research monograph publishing might be considered to be at a point of inflexion: with online distribution, fuelled by the impact of the pandemic, overtaking print, and Open Access gaining both visibility and viability, propelled by Coalition S and other research funder initiatives and policy changes.

The authors published a paper entitled ‘The Death of the Monograph?’ in *Publishing Research Quarterly* that discussed some findings from the survey and indicated that in 2021 the monograph continued to flourish as a form of scholarly communication in the arts, humanities and social sciences (Shaw, Phillips, Gutiérrez [1]). Indeed, the evidence from the survey of publishers shows that the number of monographs being published continues to grow, with commercial publishers making the larger contribution to this expansion in output. The growth in title output coincides with a reduction of dissemination in print form and explosion in digital distribution.

In this article we use evidence from the survey to investigate the state of the transition of monographs to Open Access publication. We highlight publishers’ opinions on the impact of the Covid 19 pandemic upon monograph publishing and on the future of the monograph, and the pressures and challenges faced by monograph publishers. The term monograph is used to denote a long-form academic book on a single research topic, written by a single or several authors, and, following Geoffrey Crossick [2], it also includes edited collections of research essays, critical editions of texts and other works, short form monographs and other longer outputs of research. But it excludes journal articles, textbooks and trade titles.

Much has been written over the past ten years about the application of Open Access (OA) to monographs and concerning experimentation with alternative workflows and business models. Peter Suber wrote in *Open Access* ([3], p. 107) that OA monographs may be ‘higher hanging fruit’ than OA journal articles but that they are not out of reach. He comments that authors are increasingly recognising that the wider readership and greater impact of Open Access outweigh the usually meagre royalties generated by traditional monograph sales. He also argues that both authors and publishers are noticing that for some books the existence of a full text OA edition actually boosts the sales of priced print versions.

Discussing the long form digital humanities projects funded by the Mellon Foundation in *The Academic Book of the Future,* Anthony Cond characterised the direction of travel of the academic monograph ‘… it will be digital, it will be iterative, the cost of making it available in Open Access form (if so desired) will reduce through a shared infrastructure, it will be rich in supporting data …’ ([4], p. 50).

Several writers who have looked closely at OA and the publication of monographs have commented that although the appeal of OA is abundantly clear to researchers, funders and librarians, the challenges are considerable [3–7] and [8]. Michael Jubb [7] commented, ‘Funders and policy-makers—especially in the UK—are becoming more interested in promoting OA for books, and this trend will continue. They are also aware of the challenges that have as yet prevented a more pronounced move towards OA, including costs, authors’ behaviours, rights regimes, and the complexities of the international ecology’. [7], p. 16).

Martin Paul Eve in *Open Access and the Humanities* (2014) provided a helpful summary of various studies of OA monograph publishing and discussed different economic models. He commented that ‘the economic structures for monograph production are tightly bound into editorial and gatekeeping functions, yielding a high cost to reach first copy’. He referred to ‘the dreaded steep publication charges’, saying that ‘this in turn leads to greater publisher anxiety over the long-term sustainability of a green model alongside a pay-to-purchase system’ [6], p. 136).

Schrader et al. [9] also observed that high Book Processing Charges (BPCs) are inhibiting researchers from OA publishing and for this reason recommended experimentation into alternative cost effective, personnel efficient and media neutral workflows for the publication of OA books by universities.

Ellen Collins and Caren Milloy in the *OAPEN-UK Final Report: A Five-year Study into Open Access Monograph Publishing in the Humanities and Social Sciences* [5] drew attention to the need for change in three areas: attitudes and perceptions; systems, policies and processes; and business models and warned that ‘a one-size-fits-all approach will not work for open access monographs’.

Lockett and Speicher [10] have highlighted the role of OA publishing models in stimulating the launch of new university presses. Elliot [11], reporting on work done at Emory University, drew attention to the benefits of OA monograph publishing in the humanities and described a model for university funding of OA monograph publishing in the humanities. Some studies, such as that of Schrader et al. [9], focus on the search for alternative workflow models for OA monograph publishing to enable publication by universities, perhaps implying that the publishing industry is perceived to be wedded to traditional purchase models and therefore less disposed to embrace OA.

Geoffrey Crossick in *Monographs and Open Access: A Report to HEFCE* ([2], p. 54) recognised the key role to be played by established publishers in transforming monograph publishing to an OA model, ‘Meeting the challenge of dissemination will be essential if the benefits of open access are to be secured, and one of the ways forward will be for established publishers to adopt new business models and to ensure that they are sustainable in the long run. As with peer review and brand, the behaviour of well-established publishers becomes one key element in any move towards well-disseminated open access. As they move from commodity provider to service provider, the various activities currently undertaken by print publishers will be reconfigured rather than removed.’

A central objective of the present study was to examine the current state of play in the monograph publishing industry, and to assess the extent to which publishers are engaging in Open Access monograph publishing. It was also hoped to isolate the opinions and concerns among publishers about Open Access as a direction of travel.

## The Survey Methodology

A web-based survey using the platform Typeform was developed by the team at the Oxford International Centre for Publishing at Oxford Brookes University, and was tested by several industry professionals. In total, the survey included some 60 different questions across a range of topics pertinent to the publication of academic monographs. Publishers of English language academic monographs in arts and humanities and social sciences in the US, the UK and Europe were approached in February/March 2021 with an explanatory email and were invited to complete the survey online.

25 different publishing organisations gave their responses to the survey, of which 15 were university presses, nine were commercial presses and one was a learned society.

Although this does not represent the entire universe of academic monograph publishers, the research team was confident that the responses were representative of the full range of publishers, both commercial and university press, and both large and small. Furthermore, by totalling the given 2020 output of the responding publishers the team estimated that it constituted approximately 75% of the sector’s monograph publishing output.

The data from the survey were anonymised and the results analysed. The resulting research report has been made available to participating publishers and to interested industry bodies (Shaw, Phillips, and Gutiérrez [12]).

Certain limitations apply to the survey. To preserve the anonymity of the respondents and to make the survey as easy as possible to complete, pre-set ranges were provided for some questions. This meant that for some questions it was not possible to identify precise numbers as to revenue/turnover or publishing output.

## The Impact of Covid 19

The survey was taken one year into the Covid 19 pandemic, in the first quarter of 2021. We asked the open question, ‘What has been the impact of Covid-19 on your monograph publishing business?’.

The responses to the survey show clearly that the pandemic had accelerated the trend towards increased digital distribution and the erosion of print sales. Responses from 19 out of 25 presses included a reference to the decline in print sales and to increased digital sales and/or usage. Six respondents used the term ‘acceleration’ when describing the impact of Covid upon an existing trend.

There were individual nuances in some of the responses:

- A large commercial press remarked,* ‘In 2020 we saw a dramatic shift from print to digital. Digital sales were responsible for 23% of all monograph and minor reference revenue in 2019, but 47% in 2020. Prior to 2020 we had experienced many years of growth in monograph sales, but in 2020 overall revenues for monographs dropped year-on-year (that is, the increase in digital sales did not fully compensate for the loss of print sales)’.*
- Commenting on the longer-term implications, a large university press observed*, ‘It has led to significant acceleration of migration from print to digital and we expect digital to be the dominant form of institutional access to monographs within 5 years’.*
- A smaller university press remarked on the consequences for those titles which are not available in digital format, ‘*There has been a decline in print sales, with monographs unavailable in a digital format (due to third party material) hit the hardest’.*
- One respondent commented on the underlying uncertainty about library budgets, *‘There has been an increase in e-book sales, but not enough to cover losses from library spending being put on hold as the impact of the pandemic was monitored by universities. We are awaiting more data on this’.*

Responses to this open question about the impact of Covid 19 upon monograph publishers included a range of other observations as to the effect of the pandemic on publishers’ operations. Two smaller commercial presses volunteered that the volume of proposals and manuscripts had increased, *‘more manuscripts submitted than in years past’, ‘More proposals, more author demand’.* A large commercial press commented, *‘So far, no big change in output, but of course we lost revenue in 2020’.* One university press observed,* ‘We have seen very little impact on publishing volume’.*

In contrast, several of the smaller university presses in their responses to this question drew attention to the disruptive effect of Covid upon publishing operations*: ‘We have experienced difficulties in finding readers for peer review and difficulties in acquisitions due to the cancellation of conferences and travel’, ‘starting to see more delays in typescript deliveries, which may feed through to a smaller publishing programme in 2022’,* and ‘*At pre-press stage we notice delays (authors unable to continue field work, work in the archives, library research, *etc.*), standstill, and a decline in new submissions/acquisitions.’* One of the smaller university presses offered the one-word response to this open question*, ‘crushing’.*

## Open Access Monographs

Journal publishing is well along the path of transformation to an open access model, propelled by pressure from research funders and from institutional libraries and by the emergence and establishment of a number of OA publication routes including institutional ‘read and publish’ agreements. The way forward for monograph publication, especially in the humanities and social sciences is less clear. Research funder pressure is mounting for OA publication and some of the mechanisms and publication routes are now available. The August 2021 policy announcement by UK Research and Innovation (UKRI) included the requirement for in-scope monographs published after 1 January 2024 that ‘the final Version of Record or the Author’s Accepted Manuscript must be free to view and download via an online publication platform, publishers’ website, or institutional or subject repository within a maximum of 12 months of publication’ [13].

Many monograph publishers now have mechanisms and models in place to enable OA monograph publishing on their platforms. The typical model is a ‘Gold’ author pays model requiring a Book Processing Charge (BPC), the level of which varies but is typically in the area of £8,000–£10,000.

**Fig. 1:**
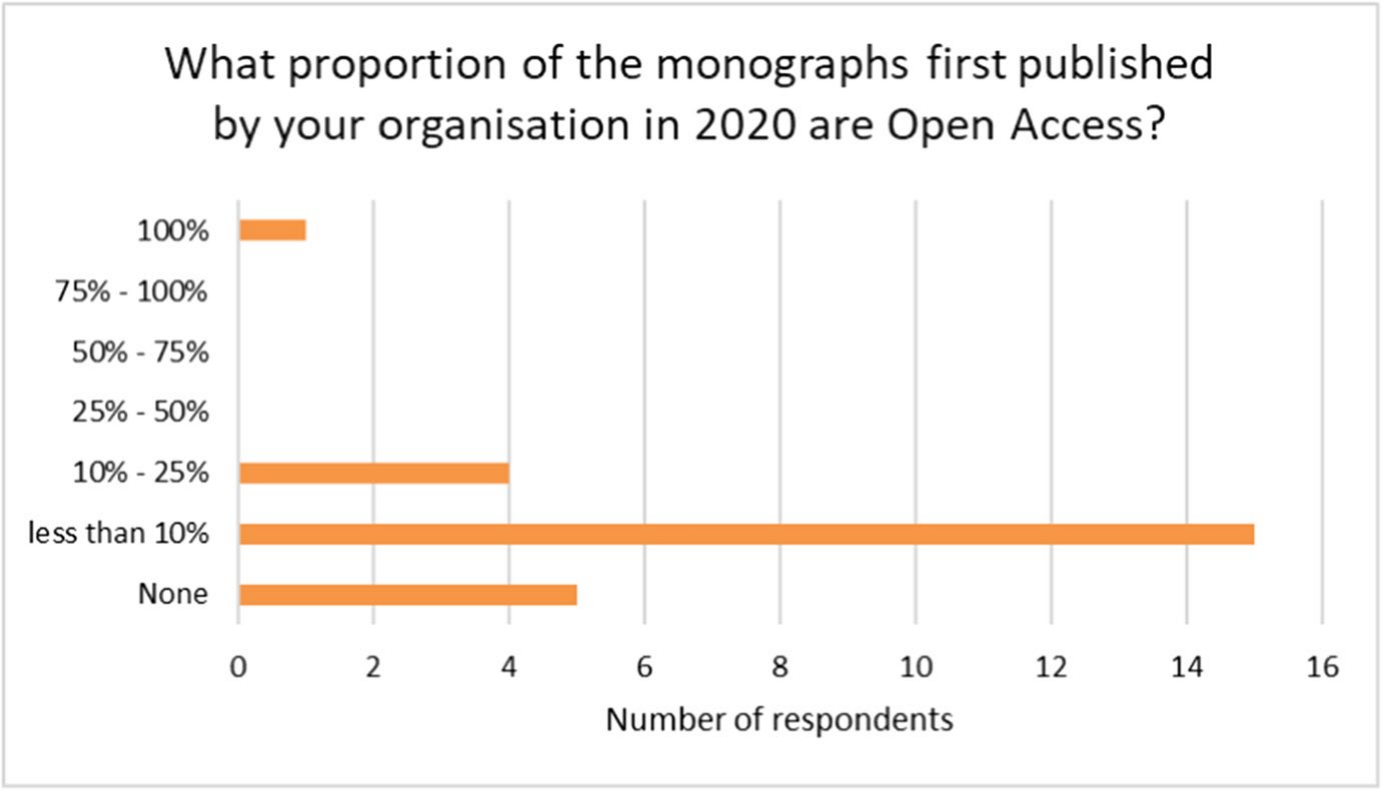
Proportion of 2020 monograph titles that are Open Access

In the survey we took soundings from monograph publishers as to the present levels of OA monograph publishing. The survey asked publishers, ‘what proportion of the monographs first published by your organisation in 2020 are Open Access?’ Although one dedicated OA publisher reported 100%, the remainder showed 25% or less as shown in Fig. 1, with the majority indicating less than 10%.

We asked respondents to describe the business model used for the OA publication of monographs. The ‘Gold’ Open Access model, typically enabled through the use of Book Processing Charges, was most frequently mentioned, with the majority of the responses that identified a model referring specifically either to the use of the Gold model (10 responses) or to Book Processing Charges/BPCs (7 responses). Three responses mentioned Knowledge Unlatched and three mentioned Green OA models, but in each case in conjunction with use of the Gold model. Six smaller university presses did not give a response to the question, of these four mentioned that they have no OA publishing model in place. One large commercial publisher referred to a discounted model available for authors of previously published titles who wish their work to be made OA.

**Fig. 2:**
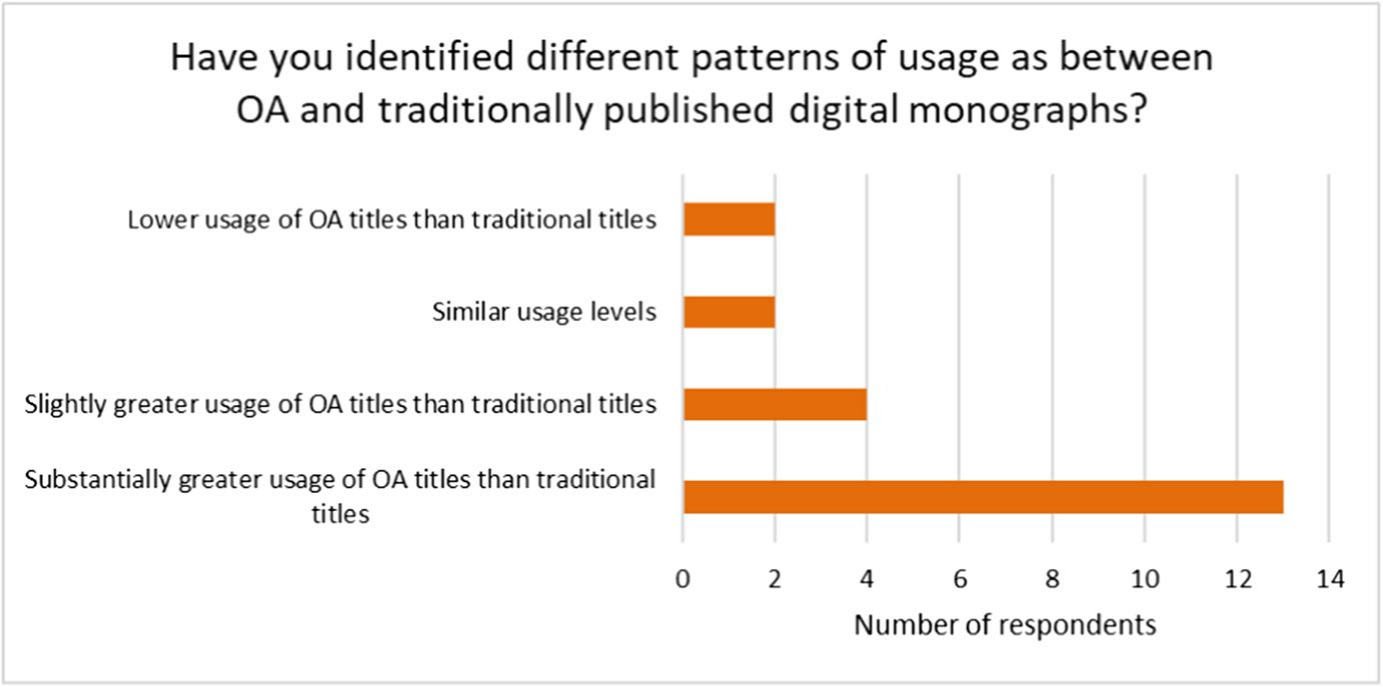
Usage patterns as between OA and traditionally published monographs

The survey showed that titles published under an OA model receive higher levels of access than titles published traditionally. The survey results indicate clearly that most publishers are seeing greater online access and usage of OA titles as compared with traditionally published digital monographs. This is shown in Fig. 2 and is illustrated by the verbatim comments that follow below.

Among the verbatim comments were several that confirm the greater usage of OA monograph content:

*‘OA [is] used more widely by a significant amount. That may result in high-quality, non-OA material being overlooked by researchers and students.*’

*‘More downloads, greater geographical spread, more social media mentions, more media coverage, more awards, more positive reader comments.’*

*‘Usage of OA monographs ranges from the thousands to the hundreds of thousands, for individual books. There is no comparison with the print editions which typically sell in the low hundreds. OA usage is also much more widespread globally. Our books have reached over 240 countries and territories worldwide.’*

One respondent commented on the wider global reach achieved through OA, *‘For academic monographs, the market for OA titles remains the same as that for print monographs. However, there is a wider global usage, including usage in countries where we would have had no reach previously’.* In a similar vein, a university press commented on the advantages of OA in bringing an author’s work to a wider readership, *‘Our recommendation to authors of monographs is to publish in OA; it is the best way to reach a global reading audience, esp. for monographs that in a traditional publishing scenario have to be marketed very actively to reach only a portion of the possibly interested audience.’*

The question on OA usage also drew some comments on the relationship between making a monograph available Open Access and the resulting impact on print sales. A large press commented, ‘*Sometimes OA seems to stimulate print usage, sometime hinders it. OA significantly reduces the use of paid-for digital formats (such as Kindle).’*

Another respondent raised the question as to how much can be inferred from the basic measurement of views of the OA text*, ‘Our data relate to views, which show a potentially significant positive [swing] to OA but it does not directly equate with engagement or value.’* This point was echoed in a comment from a large commercial press, *‘This is actually very tricky to gauge given that such a large portion of web traffic is robots. Indeed, the more niche the content, the more the “usage” is likely to be biased towards web crawlers. I hope our investments in usage analytics will help to understand this better.’*

It is clear that some publishers find it difficult to track or measure usage, either because they have only recently embarked on OA publishing, or because the information they receive from their platform or host imposes limitations, *‘It is hard for us to track as we struggle to combine the data from all the aggregators for usage of traditionally published monographs.’*

Examination of the two responses in Fig. 2 that indicated lower usage of OA titles than traditional titles reveals that the first respondent has no OA programme, the second ‘lower usage’ respondent misunderstood the question, as they offer a contradictory verbatim comment ‘…*we get reports of downloads and page views. But the volume of these activities increases dramatically for OA as compared to gated content.’*

**Fig. 3:**
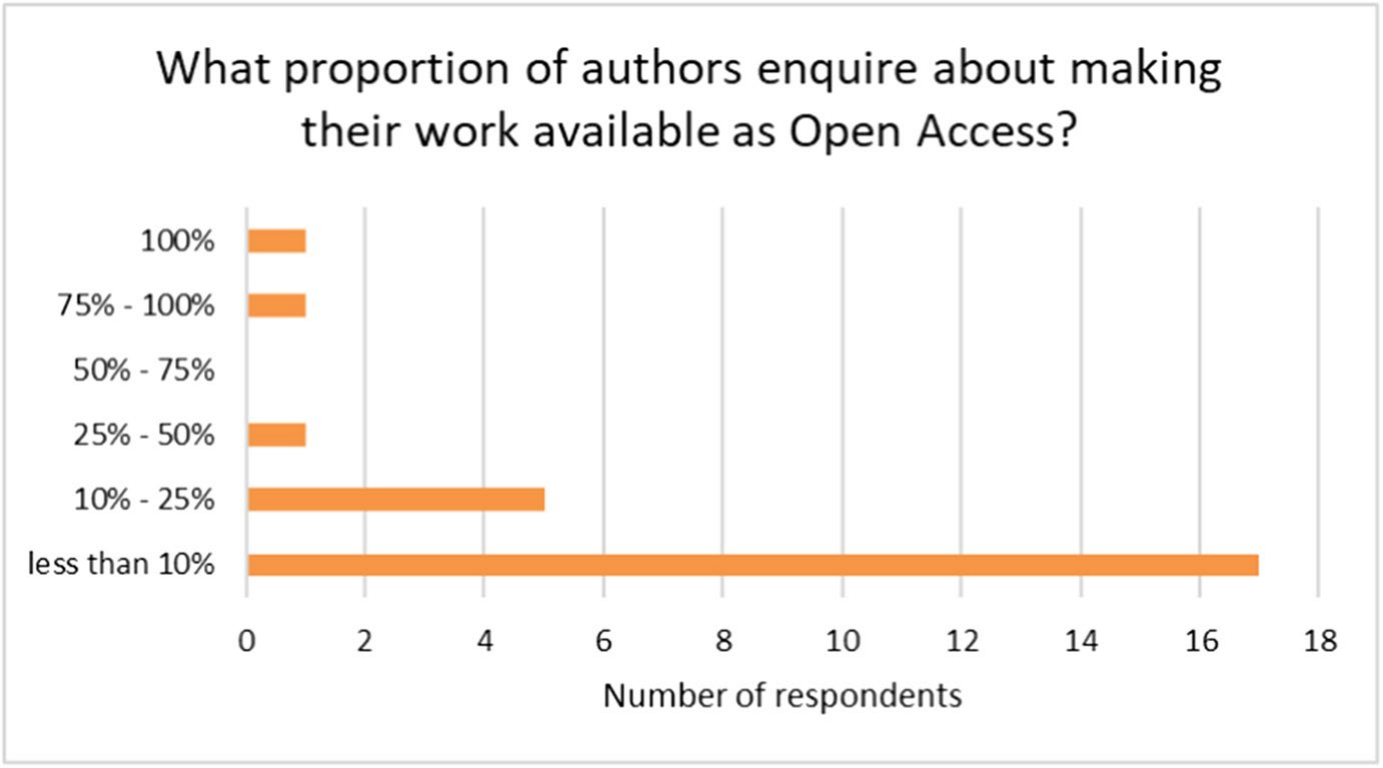
Proportion of authors that enquire about Open Access publication

The survey asked publishers what proportion of authors enquire about making the work OA. With two exceptions, the results from the survey show that publishers are receiving low levels of enquiries about OA publication of their work in monograph form. Exceptionally one university press, that offers a pure OA model and attracts authors with an interest in OA, answered 100% and a second university press indicated 75%–100%. Other than these two, publishers report that authors seldom enquire about making their monograph available as Open Access. The majority of publishers (17 out of 25) responded that ‘fewer than 10%’ of authors enquire about making their book available OA. Five other publishers (including the larger commercial and university presses responsible for the bulk of the output represented by the survey respondents) indicated that between 10%–25% enquire about OA. The results are shown in Fig. 3 below.

In a follow-up question we asked what percentage of authors in 2020 had funding for the publication of their research via Open Access.

**Fig. 4:**
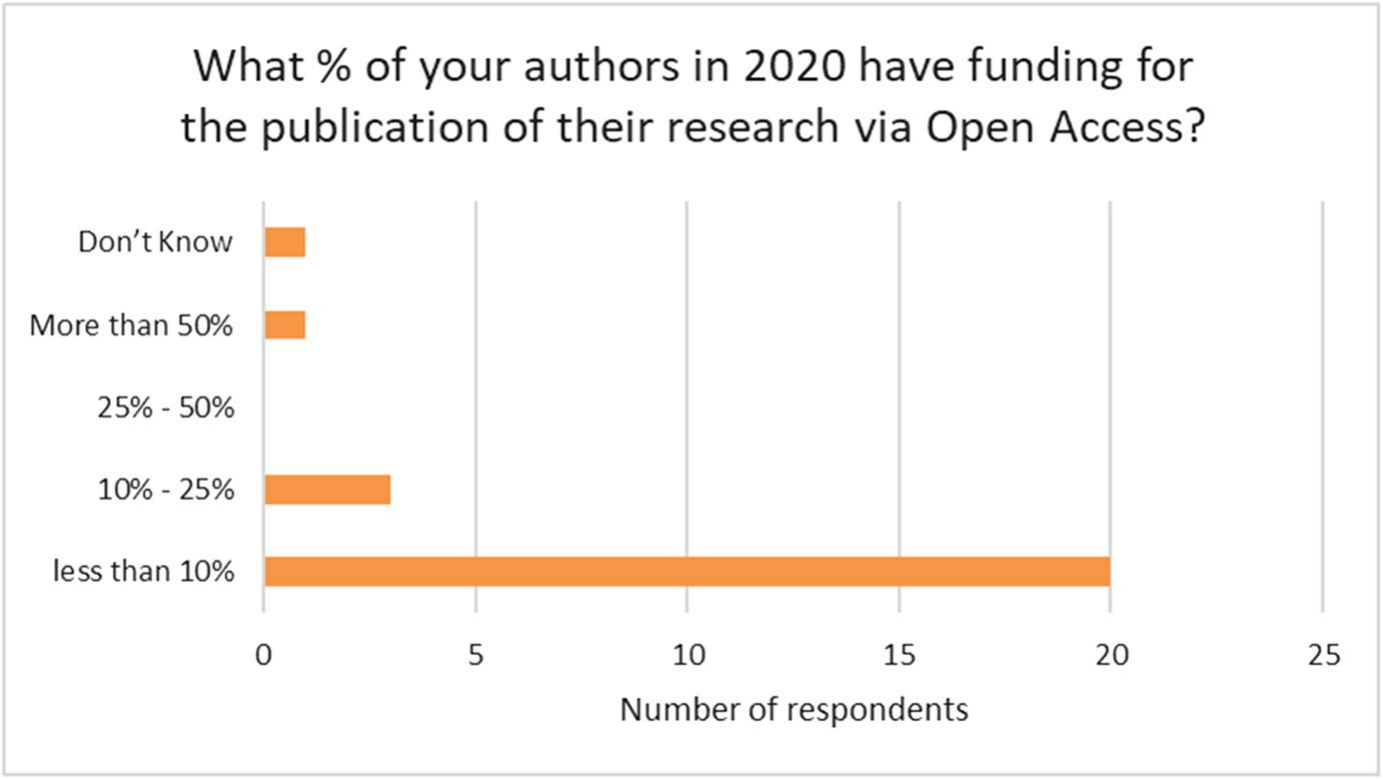
Proportion of authors with funding for Open Access in 2020

20 out of the 25 presses responded that fewer than 10% of authors have funding for OA publication. Three gave the range of 10–25% of authors with funding. One press, with an OA publishing model, indicated that over 50% of authors have funding (See Fig. 4).

**Fig. 5:**
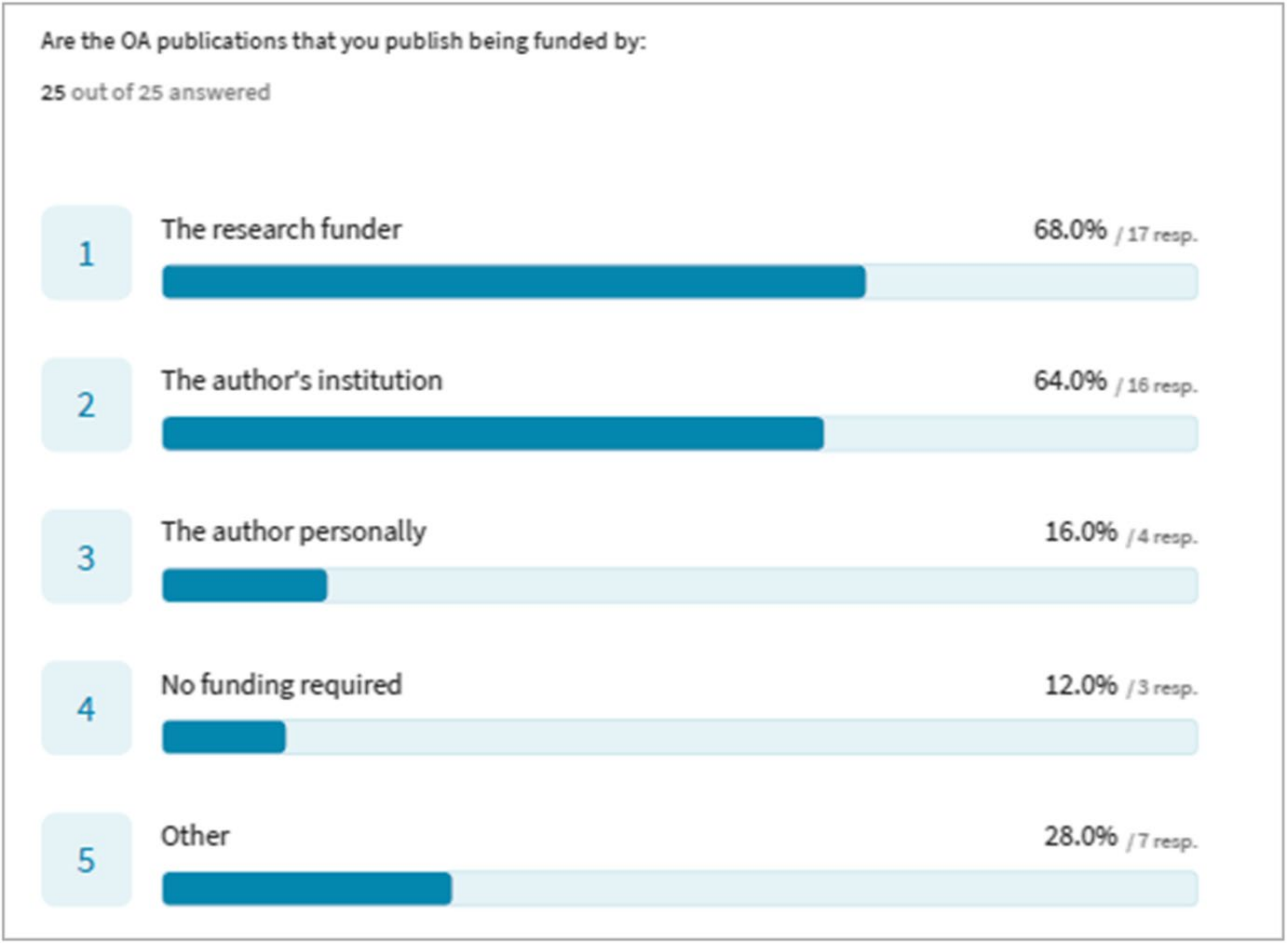
Sources of Funding for OA Publication

In a further follow-up question, respondents were asked about authors’ sources of funding for OA publication. Respondents could choose more than one option. The most frequently mentioned source of funding for OA publication was the research funder, and the second most frequent the author’s own institution (Fig. 5).

**Fig. 6:**
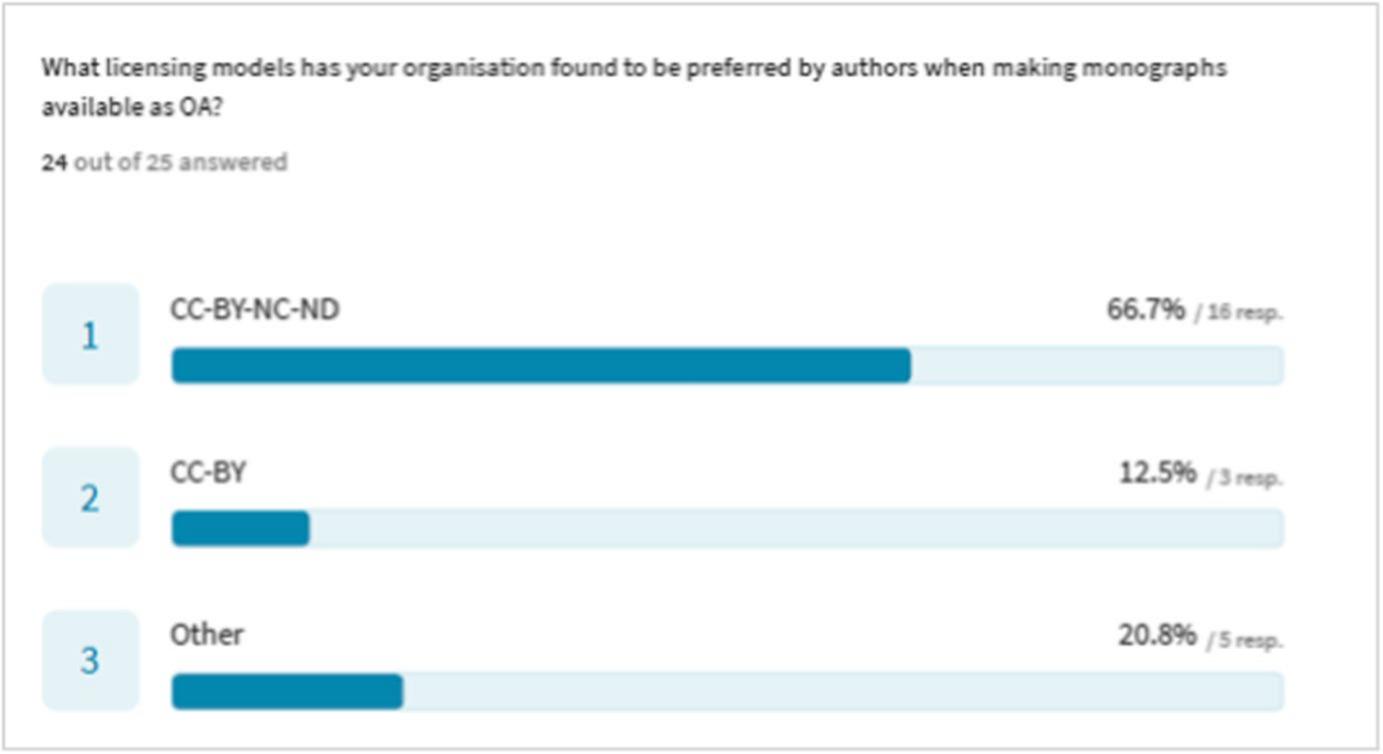
OA licensing models—author preferences

Publishers were asked about author preferences regarding the licensing model when making monographs available as OA. We asked, ‘What licensing model has your organisation found to be preferred by authors when making monographs available as OA’. 67% of respondents identified the CC-BY-NC-ND model as preferred. Of the five publishers who replied ‘other’, three undertake no OA publishing. The results shown in Fig. 6 indicate a clear preference among monograph authors for the most restrictive CC-BY-NC-ND Creative Commons licence. Further investigation into researchers’ attitudes would illuminate this area.

In exploring a related area respondents were asked, ‘What is your policy regarding authors making the text of their research monograph available through their university repository?’ The response could be made using free text and drew a wide range of answers. The variation in responses appears to show that this is an area where many different policies are applied and in three cases no policy is in place. No discernible pattern emerged, as between commercial and university presses. In general, the larger presses were able to state their policy more clearly, but the variations are considerable.

Four responses stated that they do not allow authors to make their work available in institutional repositories and one commented ‘*we discourage’*. Seven responses (including the larger commercial presses) indicated that authors may make their submitted manuscript available in their institutional repository. But of these, one applied a 24 month embargo, four presses indicated that authors could post a single chapter of the published work, and two presses allow authors to post the entire published work subject to an embargo (variously of 12 and 36 months). Three university presses were vague about precise policy but indicated willingness or flexibility to allow authors to post their work. One OA press commented that ‘*we welcome this’*.

From this brief investigation we conclude that the present range of policies may well be confusing for authors and readers. More investigation could be undertaken to establish whether authors of research monographs are confused by publishers’ policies and whether the industry might be better served by establishing a code of best practice on the posting of monograph content in institutional or other repositories.

## Clearing of Rights for OA

The clearing of rights for OA publication was identified as a problem area by 12 responding publishers, eight respondents said it was not a problem area.

We asked, ‘Has your organisation found the clearing of third-party rights and permissions to be a problem area when making monographs available as OA? What approach does your organisation take to this?’.

Among those who indicated that the area is not problematic, the following comment is representative, ‘*On the whole, no. Although we ask authors to clear these rights themselves. I do not think this issue is well understood.’*

Another commented *‘It is just an extra consideration when considering an OA route for a monograph. As a smaller part of a programme right now, it’s not a particular problem area, but could be trickier when scaled up.’*

A further comment expanded, ‘*Not especially more problematic than clearing rights for commercial publications, although there are some challenges. Some rights owners still base their charges on print runs, which doesn’t apply to OA books or books printed on demand. Some rights owners don’t grant permission in perpetuity which presents an administrative burden to have to re-clear rights every few years for occasional images. Some lenders will not agree to lend images for a book published under a CC licence. Having said this, more and more lenders are making more of their collections freely available, and increasingly lenders have fee structures for digital publishing. We aim to work with lenders to secure the permissions we need, although in some cases our approach has been to recommend to the authors that they seek alternative images if the permissions prove problematic.*’

Among the 12 that indicated that the area is a problem, several emphasised that clearing rights in order to make backlist available OA is challenging. For example, *‘It is not a problem with front list/new content. It is a huge issue for backlist content, requiring hours of research into the existing rights, trying to find an author's representative, *etc*.*’

*‘Absolutely. We communicate the issues around permissions to authors and editors early and often, for all formats of the book project (print, digital, or OA). This also limits the pool of titles we might consider for OA.’*

Several respondents referred to the removal from OA versions of materials and images for which permissions were not cleared, *‘For some books it has been tricky, but the majority of third-party rights holders are familiar with OA licensing. If permission has not been secured for the material, then the material is removed from the OA edition.*’

Another commented, ‘*It's a problem to clear image rights (in particular of museums). We sometimes publish the OA version with no image (provided it is not necessary for understanding the text).*’

An interesting insight was offered by a commercial publisher*, ‘In general we find it is somewhat harder to secure permissions for re-use of third-party material in open access books. Our preference is for authors to secure permission for third-party material in OA books to be released under the same Creative Commons licence as the rest of the book. As rights holders can sometimes be reluctant to agree to this, if the third-party material is integral to the work, we will permit it to be included ‘all rights reserved’ (i.e. excluded from the CC licence). This helps in many cases. However, in some areas it remains extremely difficult to secure permissions for inclusion in OA works, notably where the rights are held by commercial organisations (e.g. fashion marques, other brands, major film studios), and in some cases where they are held by GLAM organisations (Galleries, Libraries, Archives, Museums).’*

## Metadata and Ensuring Awareness of OA Publications

The dissemination of and creation of awareness of OA monograph content have previously been mentioned as challenges to the OA publication of monographs [2]. Interestingly, the survey of publishers indicated that these are no longer considered by most publishers to be major problem areas, solutions having been found to the challenges.

In an open question we asked, ‘Dissemination of OA research monographs has been identified as a particular challenge What measures does your organisation take to maximise the awareness of OA monographs?’.

Respondents’ replies split into three broad categories:

- Those for whom the question was not relevant as they are not actively publishing OA monograph titles (five responses).
- Seven university presses and one commercial press who remarked that the problem raised in the question no longer exists, pointing to the various platforms and routes for dissemination (such as DOAB, OAPEN, Project Muse and JSTOR) and to the marketing efforts applied to OA titles which are equivalent to the marketing of other monographs.

One especially comprehensive response from a large university press summarised this perspective, ‘*The Crossick Report is now 6 years ago so it is worth noting that awareness and expectations of funders, institutions, and researchers as both authors and information users have changed. However, all our OA monographs are discoverable and can be found as an open category on our platform. We deposit in Google Books and DOAB and the DOI record indicates that the work is available with an open licence.’*

Another response similarly lists the actions taken to address the need, *‘We have our own open access publishing platform, …We also distribute our OA titles *via* OAPEN, and *via* Knowledge Unlatched when funding [is]available. We ensure our titles are included in DOAB. We include OA metadata in the MARC records available for our titles from the publishing platforms.’*

(c) Commercial presses and some university presses who, in a range of responses identified or described the marketing efforts made to raise awareness (nine responses). Such marketing efforts ranged from: ‘*Same marketing as print books—newsletter, social media, conferences, flyers, launches’,* to ‘*All of our Editors are trained to speak about this Topic… We publish White papers on OA publishing’*, and ‘*For awareness, authors are always the best promoters. We make sure they understand they can use the text itself to promote the book’*. One commercial press added the comment *‘…we also make the Kindle versions available for free.’*

Although, as noted, several university presses did not recognise any challenges in dissemination or in raising awareness of OA books, one large university press commented that ‘*We expect this aspect of book publishing to develop considerably in the coming years.’*

Whereas ensuring readers’ awareness of Open Access monographs is not considered by most publishers to be a challenge any longer, the related area of standardisation in the use of metadata across the industry for content dissemination was raised by several responses to the survey.

We asked for free text responses to the question, ‘Are there areas of technology shortfall where, in your view, the available technology solutions lag behind the expectations of authors, readers or publishers for the publication or dissemination of research monographs?’ The responses ranged across a variety of different topics, and seven respondents highlighted metadata standards and dissemination as an area of perceived technology shortfall. Among the verbatim comments were:

*‘Metadata collection and dissemination (ongoing improvement, driven in part by Open Research). Content discovery and access.’*

*‘Creation and distribution of metadata is an ongoing challenge. Although there are established industry standards such as ONIX and MARC, virtually all third-party distributors, discovery tools and other intermediaries require their own versions of these standards (if not their own proprietary data standard). Creating these metadata feeds and delivering them is a challenge for small and medium-sized publishers.’*

*‘Also, the process by which metadata is enhanced and distributed via the online channels is opaque, and the analysis work to identify where errors have occurred and resolve these issues absorbs a large amount of time.’*

A large commercial press commented*, ‘Bibliographic and metadata management systems: Such systems for books (which tend to be used by publishing staff rather than authors) are often cumbersome to use and difficult to update. Where such systems are used by trade and academic presses alike, they are not always well adapted to academic needs, and particularly to open access, and development queues can be long, meaning manual workarounds are needed.’*

*‘Open access policies increasingly require that action be taken at chapter level—that is, a funder may require that a chapter in an edited work that results from their grant funding be made open access. However, at most stages of the book supply chain, including in-house metadata management and dissemination via third parties, systems are not well adapted to manage or differentiate content at chapter level.’*

## Conclusions on OA

Drawing together the Open Access monograph strands, it is apparent that the industry is at the early stages in the adoption of Open Access (OA) models of monograph publishing. Although usage of OA monograph content is higher than that of conventionally published online monographs, less than 10% of publishers’ output is being made available OA and fewer than 10% of authors are asking for their work to be made available open access. Factors that may be restraining the take-up of OA monograph publishing models to date may include: the relatively high cost of gold (author pays) OA models, the lack of funding for OA publication in the HSS disciplines, the absence of applicable read and publish models, concern regarding the CC licence, or simply the lack of awareness. More research is needed to form a clearer understanding of the restraining factors and to identify possible solutions. That said, it is to be expected that research funder directives such as UKRI’s August 2021 statement that from 2024 all monographs with funding from UKRI must be made available through an OA model will lead to change in author requests for OA publication at least in respect of many UK authors [13].

In the survey publishers were asked ‘Given the current direction of travel, do you expect your organisation to be publishing monographs in ten years time?’ In reply, all 25 respondents confirmed that they expect to be publishing monographs in ten years’ time but with many expecting the format and/or the model to change.

A large commercial press commented*, ‘Yes, but it is likely to look dramatically different. We anticipate that in ten years’ time a much larger proportion of the monograph list will be published open access, and that new models will be necessary to manage this transition in a sustainable way.’*

Several respondents commented that the format, or the ‘container’ may be different in ten years’ time. A medium sized commercial press said, ‘*Unsure of the containers content will live in (i.e. whether they would still be labelled monographs, will there be a distinction between books or journals), but we intend to continue publishing impactful content for many years to come’.* And a university press indicated, *‘We expect that monographs will still be published, but the main edition is likely to be the digital format. What that format will be is still open to question. Print editions will still be made available using the print on demand model.’*

Another university press expressed confidence in the persistence of long form monograph publishing*, ‘Whilst monographs in ten years' time might not be in the form recognised today, I do believe we will be publishing long-form pieces of writing and research (i.e. the monograph) in whatever format (in print, e-book or other) as long as this is what is required by the academic community who[m] we serve. If it is not required, then we will adapt to their needs to provide the quality publications they do require in the future.’*

We can see that although the monograph is expected to continue, the form and the business model are much less clear. Most monograph publishers are now offering Open Access and anticipate that it will continue to grow, but several major presses qualified this expectation with observations about the need for direct funding for OA publication, and expressed concerns about research funder requirements for Green OA access. The parallel availability of print-on-demand versions of online Open Access monographs is likely to be a continuing feature of the landscape, but without first copy costs underwritten by gold open access BPCs it is unlikely that such a model would ensure the economic viability of many OA monographs.

Among the survey comments as to whether their organisation might be expected to be publishing monographs in ten years' time a large university press remarked *‘Hopefully. They are a core part of our publishing and offer a distinctive value in AHSS research. Whether monograph publishing is sustainable depends on whether OA policies insist on Green OA access. We do not believe that the current publisher investment in long form research is sustainable without a funded solution.’*

Another substantial university press remarked, ‘*We hope so. Green OA mandates from funders are of considerable concern to us though. We need scalable, sustainable approaches to Gold OA for research monographs’.* A third, smaller university press offered a similar observation, *‘Yes, it will still be viable, but it will continue to be financially challenging. Current trends of scholars using digital for discovery and print for in-depth reading suggest the model will continue to be simultaneous. A shift to direct funding rather than [a] cost-recovery model would be ideal, and allow us to make the shift fully to OA, but those dollars seem not to be available in the amounts needed within the US scholarly ecosystem—at least for HSS monographs published by university presses.’*

The survey demonstrated clearly that most publishers see monograph publishing in the humanities and social sciences as being in transition, with Open Access expected to play a key part in the future. In can be inferred that publishers expect a mixed economy of OA and ‘toll-access’ monographs to continue, with both being hosted in online collections and sold individually as hard copies printed on demand, at least in the short run. Those publishers who are successful in bringing down the costs of monograph publication and in achieving economies of scale in their publishing operations, while maintaining editorial quality, may be able both to command a larger market share among OA authors and to offer toll-access customers greater critical mass in their collections.
